# Bayesian Network Applications for Sustainable Holistic Water Resources Management: Modeling Opportunities for South Africa

**DOI:** 10.1111/risa.13798

**Published:** 2021-08-02

**Authors:** Indrani Hazel Govender, Ullrika Sahlin, Gordon C. O'Brien

**Affiliations:** ^1^ Department of Horticulture Durban University of Technology Durban South Africa; ^2^ Centre for Environmental and Climate Science (CEC) Lund University Lund Sweden; ^3^ School of Biology and Environmental Sciences, Faculty of Agriculture and Natural Sciences University of Mpumalanga Nelspruit South Africa

**Keywords:** Bayesian networks, water resources, South Africa

## Abstract

Anthropogenic transformation of land globally is threatening water resources in terms of quality and availability. Managing water resources to ensure sustainable utilization is important for a semiarid country such as South Africa. Bayesian networks (BNs) are probabilistic graphical models that have been applied globally to a range of water resources management studies; however, there has been very limited application of BNs to similar studies in South Africa. This article explores the benefits and challenges of BN application in the context of water resources management, specifically in relation to South Africa. A brief overview describes BNs, followed by details of some of the possible opportunities for BNs to benefit water resources management. These include the ability to use quantitative and qualitative information, data, and expert knowledge. BN models can be integrated into geographic information systems and predict impact of ecosystem services and sustainability indicators. With additional data and information, BNs can be updated, allowing for integration into an adaptive management process. Challenges in the application of BNs include oversimplification of complex systems, constraints of BNs with categorical nodes for continuous variables, unclear use of expert knowledge, and treatment of uncertainty. BNs have tremendous potential to guide decision making by providing a holistic approach to water resources management.

## 1 INTRODUCTION

Water resources are under threat globally due to anthropogenic transformation of land and the growing human population, resulting in unsustainable consumption of natural resources and loss of biodiversity (Naiman & Dudgeon, 2011; Vörösmarty et al., 2010). This has impacted on water availability to sustain aquatic habitats and human requirements for basic needs. Water resources management is subject to complexities and uncertainties that are framed within a temporal and spatial context. The spatial context lends itself to a range of threats and stressors, arising from land‐based activities interacting through various pathways, impacting on socioecological systems (Allan & Johnson, 1997; Fuller & Death, 2018). The threats posed by the plethora of land‐based activities are increasingly analyzed using risk‐based approaches to examine synergistic effects of multiple stressors (Landis, 2021; O'Brien, Dickens, Baker, Stassen, & van Weert, 2020; Page et al., 2012; Sperotto et al., 2019). In water resources management, the complexity of source, stressor, receptor, and endpoint variability and stakeholder values need to be addressed in a holistic approach. This holistic approach should consider connections between different components of a system to provide an overview of the well‐being or health of an entire socioecological system. Models have been developed to represent ecological risks of multiple stressors in a holistic context, at different spatial scales, and incorporating multiple levels of biological organization, varying from species level to habitats, ecosystems, and complex catchments (Leuven & Poudevigne, 2002).

South African water resources management advocates the holistic consideration of multiple drivers that affect our variable, vulnerable water resources (Dickens, Smakhtin, McCartney, O'Brien, & Dahir, 2019). The growing human population in South Africa (Zubaidi et al., 2020), expanding urban areas and water requirements for agriculture and industry, has increased the demand for the use of water resources (King & Pienaar, 2011; O'Keeffe, 2012). Factors contributing to limited availability of water resources in South Africa include irregular rainfall in some parts of the country, resulting in variable water availability across the landscape, extreme weather events, and regular drought (El Chami & El Moujabber, 2016; King & Pienaar, 2011). Compounding these issues are food security, poverty, and lack of access to basic services throughout the country (King & Pienaar, 2011). Uncertainties associated with the impacts of climate change, including a possible decrease in rainfall in the interior of South Africa and more frequent droughts and flooding in vulnerable areas, increase uncertainty to sustainable water resources management (Kusangaya et al., 2014). This demand for water supply to society is in competition with water required by aquatic ecosystems to sustain natural processes (O'Keeffe, 2012). All of this uncertainty associated with the present and future variability of water resources and increasing demands and threats to sustainability intensifies the need for effective water resources management, using good international practices and robust tools and techniques to guide sustainable development and conservation of water resources in South Africa. Through the application of a holistic approach to manage water resources in complex socioecological systems at a regional, landscape, or catchment scale, there may exist opportunities to achieve a degree of balance between protecting water resources and providing for society (Hope, 2006; Leuven & Poudevigne, 2002).

Risk deals with two issues interacting: (i) probability of an adverse event occurring and (ii) the consequence of the adverse effect (Leuven & Poudevigne, 2002). According to the “South African Ecological Risk Assessment Guidelines,” ecological risk assessment (ERA) may be defined as “the process that evaluates the likelihood that adverse ecological effects may occur or are occurring as a result of exposure to one or more stressors” (Claassen, Strydom, Murray, & Jooste, 2001; Murray & Claassen, 1999; O'Brien & Wepener, 2012). ERA) determines potential impacts of stressors to specific endpoints. Endpoints were originally specific organisms at risk of adverse effects from contaminants in the environment, but as ERA developed, more complex systems were assessed using ERA protocols to determine potential risks to multiple endpoints (Ayre & Landis, 2012). ERA has played a significant role in the United States (Hope, 2006; Suter 2008). This approach has the potential to substantially contribute toward the effectiveness and efficiency of management of the balance between the use and protection of aquatic ecosystems in South Africa (O'Brien & Wepener, 2012).

Globally, ERA, using the relative risk model (RRM) (Landis & Wiegers, 2007), has been applied extensively to landscape scale studies (Kanwar, Bowden, & Greenhalgh, 2015; Landis, 2003; Zhao & Zhang, 2013). Over time, there has been a trend toward incorporation of Bayesian networks (BNs) to existing ERA frameworks, to improve the modeling capabilities and add value as a decision support tool (Ayre & Landis, 2012). The National Water Act No. 36 of 1998 (NWA, 1998) and the South African National Water Resources Strategy recognize catchments as complex socioecological systems and prioritize the holistic management of aquatic ecosystems, by advocating integrated water resources management (IWRM) (DWA, 2013). IWRM research has also demonstrated the value of BN modeling, through supporting decision making and the ability to integrate with other models (Barton, Saloranta, Moe, Eggestad, & Kuikka, 2008; Bromley, Jackson, Clymer, Giacomello, & Jensen, 2005; Castelletti & Soncini‐Sessa, 2007). According to Kaikkonen, Parviainen, Rahikainen, Uusitalo, and Lehikoinen (2021), BNs are not fully explored in ERA applications. BNs allow for a holistic approach that considers multiple stressor and socioeconomic impacts, which has been a significant omission in the past in risk‐based water resources management. Back in 1999, Batchelor and Cain pointed out the potential of BNs as a “a powerful tool for simulating the interactions between physical, social and economic variables.” Hence, in a period spanning more than 20 years, the holistic application of BNs has not been fully explored. This article explores the characteristics of BNs, which make them suitable for application in guiding decision making, and regulation of water use and water protection measures in the South African context. The significance of BN applications to South Africa's legislated holistic water resources management approach is assessed against the available literature on BN applications in water resources management.

## 2 BAYESIAN NETWORKS

### 2.1 General Overview of BNs

BNs were first named by Judea Pearl in 1985 (Pearl & Mackenzie, 2018). BNs consist of qualitative components and quantitative components (Aguilera, Fernández, Fernández, Rumi, & Salmerón, 2011). The qualitative components indicate the dependencies and independencies between variables in the system, while the quantitative components indicate the strength and the nature of the dependencies between variables. A BN consists of nodes, representing system variables, and arcs or connectors, which represent the dependencies or relationships between the nodes (Phan, Smart, Capon, Hadwen, & Sahin, 2016). BNs represent complex systems visually, making probabilistic dependencies or causal pathways explicit. A BN can be informed by combinations of expert judgment (e.g., based on the literature or structured expert knowledge elicitation), data from empirical studies or surveys, or simulation models (Marcot, 2012; Marcot, Steventon, Sutherland, & McCann., 2006). Depending on the information used, a BN can be trained (parametrized) using more or less algorithm‐based approaches. A BN expresses the joint probability distribution over variables in the system that is being studied. By placing the variables in a network corresponding to conditional dependencies, it is possible to construct the joint probability distribution from marginal and conditional probability distributions (McCann, Marcot, & Ellis, 2006). Thus, it is possible to link variables from different parts of the system without having joint data sets for all of these.

The original BNs by Pearl were networks for categorical nodes informed by expert judgment. Today, BNs include different types of models, ranging from networks with categorical nodes to continuous state nodes, or data‐driven networks, used for the purpose to derive a likelihood for data, to Bayesian belief networks, used for probabilistic reasoning (Koller & Friedman, 2009; Sahlin, Helle, & Perepolkin, 2021). Generalizing a BN to a probabilistic graphical model has the consequence that any Bayesian statistical model can be seen as a BN because they are a joint probability distribution over a network of variables and parameters (also known as the directed acyclic graph) (McElreath, 2018). A probabilistic risk assessment model defining a probability distribution for inputs and outputs can also be a BN with latent functional relationships behind the conditional probabilities (Carriger & Barron, 2020)

**Fig 1:**
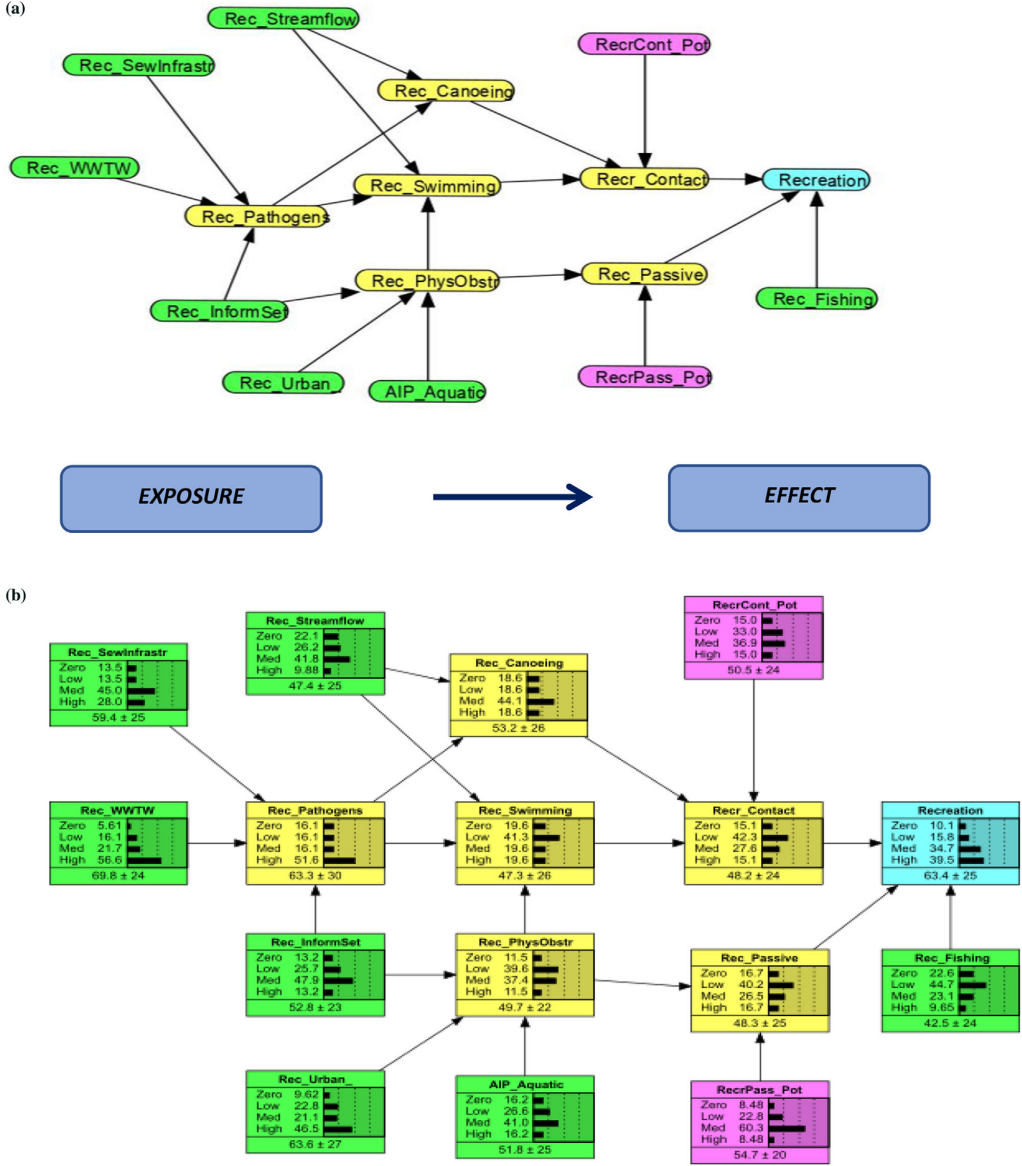
An example of a conceptual model (a), highlighting the exposure and effect pathway representing a socioecological system, where risk to recreational water use is assessed.

The example in Fig. 1 represents a system where risk to recreational water use is being assessed. The three different types of recreational use are fishing, contact recreation, and passive recreation. The color representation is explained below and makes the causal links explicit. Each variable is discretized into four states, Zero, Low, Medium, and High, based on a ranking scheme used traditionally in the RRM and subsequently in the Bayesian network RRM (Hayes & Landis, 2004; O'Brien, Dickens, et al., 2018; O'Brien & Wepener, 2012). The variable states are representative of the degrees of impact or condition of the measurable entity. Zero represents no impact, pristine, or close to reference conditions; Low represents low impact, or close to pristine; Medium represents moderately impacted/ moderately modified; High represents highly impacted. In this example, the probability of risk to recreation is predominantly in the high state, indicating that the risk to recreational water use is high, conditional on the data and information provided in the model.

Green = Exposure Parent node; Yellow = Exposure Child node; Pink = Effect Parent node; Blue = Effect Child/ Endpoint node. The conceptual model (a) is translated into a Bayesian Network (b). The variables are discretized into ordered risk categories, namely, zero, low, medium, high risk for each node.

### 2.2 Benefits of BN Applications

There are a number of merits for the application of BNs to a range of environmental problems. BNs are capable of representing complex systems consisting of a large number of variables, with the ability to facilitate the prediction of possible consequences (Aguilera et al., 2011; Uusitalo, 2007). Due to the probabilistic nature of BNs, they have the potential, if used well, to quantify risk or uncertainty (Sahlin et al., 2021). BNs are not limited to one type of information and can integrate data and scientific evidence as well as expert knowledge and stakeholder inputs. BNs are therefore suitable for modeling in ecological assessment, even when data may be missing or limited.

Another advantage is that BNs make it possible to make inference on any node(s) in the network, conditional on all other nodes. Hence, it is possible to make inferences regarding the state of a system node based on observed or assigned states on variables dependent on this node. BNs are useful to support assessment and planning using scenarios or associating nodes with utility, costs, or benefits. The graphic character of BNs makes it easy to visualize the model to experts and nonexperts, supportive in stakeholder collaboration and partnerships, enabling interdisciplinary discussions and input (Batchelor & Cain, 1999; McCann et al., 2006). BNs facilitate interdisciplinary conceptual modeling with multiple kinds of data, which is fundamental to holistic water resources management.

## 3 METHOD

This perspective article includes a review of the literature to determine the extent to which BNs have been applied to water resources management in complex systems, adopting a holistic approach globally and in South Africa. The literature was searched using Scopus, Web of Science and Science Direct. Searches were undertaken, using the key terms “Bayesian network,” “ecological risk assessment,” “South Africa,” “water management,” and “holistic,” in different combinations. The results were screened using the following criteria for inclusion as valid results relevant to South Africa:

- study employing a formal risk assessment framework,
- application of BNs,
- relevance to water resources management, and
- relevance to South Africa.

Coastal and marine focal areas were excluded from the selected literature. Although the focus was to determine the extent to which BNs are applied in water resources management in South Africa, selected publications were reviewed to support an overview of the global applications of BNs in water resources management, whereby opportunities and lessons learnt can inform application of BNs in South Africa. This was not intended to be a comprehensive, systematic review of the literature pertaining to application of BNs in ERA of water resources management. A further coverage of the literature to support the sections to follow, pertaining to BN applications, included peer‐reviewed publications and gray literature gathered over the period 2017–2021, which was intended to achieve a deeper understanding of BN applications globally.

## 4 RESULTS

Using the search term “Bayesian network ERA water resources,” Scopus generated 11 publications and Web of Science generated 12 publications. The same search term using Science Direct resulted in 675 results, and 599 of these were published from 2010 to present. Adding the terms “holistic,” resulted in both Web of Science and Scopus turning up two publications, namely, O'Brien, Dickens, et al. (2018) and Vezi, Downs, Wepener, and O'Brien (2020). The same application to the Science Direct search resulted in 146 publications, of which only two publications met the criteria above (Vezi et al., 2020; Wade, O'Brien, Wepener, & Jewitt, 2021). Table I summarizes the literature identified that is relevant to BN application is South African water resources management. A list of the publications resulting from the searches above is available as Supplementary Information. Three significant peer‐reviewed publications meeting the above criteria were not detected in the search (Agboola, Downs, & O'Brien, 2020; O'Brien et al., 2020; O'Brien, Smit, & Wepener, 2021).

**Table I:** Literature Results for South African Applications of BNs (Including Gray Literature)

**Author**	**Title**	**Date**	**Focus**	**Method**	**Peer Reviewed (J) / Gray Literature (G)**
O'Brien et al.	Regional Scale Risk to the Ecological Sustainability and Ecosystem Services of an African Floodplain System.	2021	Multiple stressor ERA—ecosystem services	BN relative risk model	J
Wade et al.	Risk Assessment of Water Quantity and Quality Stressors to Balance the Use and Protection of Vulnerable Water Resources.	2021	Multiple stressor ERA in the legislative context	BN relative risk model	J
Agboola et al.	Ecological Risk of Water Resource Use to the Wellbeing of Macroinvertebrate Communities in the Rivers of KwaZulu‐Natal, South Africa.	2020	Multiple stressor ERA—risks to biota	BN relative risk model	J
O'Brien et al.	Sustainable Floodplains: Linking E‐Flows to Floodplain Management, Ecosystems, and Livelihoods in the Sahel of North Africa.	2020	Holistic regional environmental flows	PROBFLO	J
Vezi et al.	Application of the relative risk model for evaluation of ecological risk in selected river dominated estuaries in KwaZulu‐Natal, South Africa.	2020	Multiple stressor ecological risk assessment—estuaries	BN relative risk model	J
O'Brien, Dickens, et al.	A regional‐scale ecological risk framework for environmental flow evaluations.	2018	Environmental flow assessment to guide water allocation	BN relative risk model	J
Wepener et al.	Linking Land Use to Water Quality for Effective Water Resource and Ecosystem Management	2015	Multiple stressor ERA in the legislative context	Relative risk model with BN application	G
Dabrowski et al.	Linking Land Use to Water Quality for Effective Water Resource and Ecosystem Management	2013	Link between land use and water quality	BN applications for decision making	G

In a review publication by Phan et al. in 2016, it was found that, of 111 publications that were reviewed, only three publications were studies conducted in Africa, up to that point. A further review of the literature, within the scope of this article, revealed that BNs have not been commonly applied to water resources management in South Africa. However, this trend has started to change in recent years, with unpublished studies also applying BNs to water resources management (Dabrowski et al., 2013; O'Brien, Dickens, et al., 2018; Vezi et al., 2020; Wepener, Dlamini, O'Brien, & Malherbe, 2015). Dabrowski et al. (2013) and Wepener et al. (2015) were unpublished research reports; hence, these were not picked up on research databases. This may be a limitation when conducting systematic reviews or comprehensive literature reviews, as many credible, scientific reports are excluded, despite their scientific sound methodology and findings. Phan et al. (2016) concluded that application of BNs to water resources management in developing countries should be prioritized, especially in countries where data were often limited. In addition, the authors of the same publication pointed out the significance of evaluating options as adaptive responses to climate change.

## 5 BAYESIAN NETWORK OPPORTUNITES TO MODEL SA WATER RESOURCES MANAGEMENT

There are a range of tools and methods used to model water resources. These include hydrological models incorporating stream flow data and groundwater recharge, models with a GIS interface, and a number of adaptations to northern hemisphere models for application to the African context (Hughes, 2016, 2019; Hughes & Mazibuko, 2018). Each of these has their merits; however, this article is limited to the application of BNs in dealing with water resources management. BNs have been applied to water resources management since the late 1990s (Varis & Kuikka, 1997). Research focusing on managing water resources using BNs in ERA have been conducted globally in the USA, Europe, and Australia (Ayre & Landis, 2012; Bromley et al., 2005; Crossman & Pollino, 2018; Sperotto et al., 2019). These case studies have various lessons for South Africa to learn from. Many of these are expanded further in the discussion that follows.

A BN can be constructed to update the probability distribution of system states or make predictions, as new data or evidence becomes available. Managing natural resources and ecological systems requires input from various stakeholders, scientific experts, and the relevant authorities to ensure that decision making is effective. Water is a precious natural resource, which is valued differently by various stakeholders. BNs are suitable for modeling systems with multidisciplinary input and therefore appropriate for risk assessments (Hart & Pollino, 2008). Where discipline‐specific data or information is missing, this can be used to update the model as new research is conducted or as new projects are concluded. Where resources are limited when collecting data, thus preventing successful execution of monitoring programs, BNs would provide a practical method of using limited data, together with expert knowledge to provide a more accurate interpretation of variable relationships in a system. This would be suitable for the South African context where data deficiency due to infrequent or irregular monitoring and maladministration may prevent the generation of complete data sets for training a model (STATSSA, 2019).

Owing to the capability of BNs to represent complex spatial interactions between system variables, they are suitable for holistic application to catchment scale studies (Ayre & Landis, 2012). BNs can be integrated with GIS that makes spatial dependencies explicit, facilitating visualization of causal relationships (Celio, Koellner & Grêt‐Regamey, 2014; Moe, 2010), e.g., to model alternative scenarios for forest conservation applied to water resources management (Gonzalez‐Redin, Luque, Poggio, Smith, & Gimon, 2016). BNs have tremendous potential to produce models that allow decisionmakers to weigh a number of different scenarios or management options against each other. This provides foresight to predict the possible trade‐offs in selecting one option over another, based on sound scientific practices (Schmitt & Brugere, 2013; Xue, Gui, Lei, Sun, et al., 2017).

The integration of hydrological and land use modeling using BNs has huge potential for land use planning processes as this makes alternate scenarios visually explicit, using mapping tools and geodata (Celio et al., 2014). It is also suitable to model temporal scenarios using BNs. This could be potentially useful in screening development applications in South Africa, where there is a regulated environmental impact assessment (EIA) process (DEA, 2017). BNs have the potential to provide management alternatives, based on different scenarios (Agboola et al., 2020; Vezi et al., 2020). Once a network has been constructed for a specific case, this basic structure provides the framework for different data inputs. This has application for the EIA process, whereby proposed developments can be screened using different data sets or variations to the basic BN model structure representing the ecosystem, to depict different management scenarios. With advances in models that represent ecosystems, the ability to predict change is increasingly used to guide decision making.

## 6 SOUTH AFRICAN WATER RESOURCES MANAGEMENT FRAMEWORK

The South African National Water Act No. 36 of 1998 (NWA, 1998) recognizes the need to use water sustainably to benefit society, for both current and future generations, while protecting water resources to ensure continued ecological function. Integrated management of water resources is advocated at a regional or catchment level where required, as this would facilitate a more meaningful participation by society. The NWA sets out to ensure protection, use, development, conservation, and management of South African water resources, to facilitate social and economic development. This may be achieved by providing for the increasing water demands, and protection of biodiversity, through prevention and reduction of water resources degradation. Historically water resource use was permitted without consideration for the broader catchment‐wide issues (King & Pienaar, 2011). The National Water Act (NWA, 1998) now includes a chapter on resource‐directed measures (RDMs) that includes the requirement for the minister of the Department of Water and Sanitation to: (i) determine the ecological reserve (synonymous with environmental flows), classify the water resources using the Water Resource Classification System (WRCS), and establish resource quality objectives (RQOs) on appropriate catchment or water management area (WMA) spatial scales in South Africa (Dickens et al., 2019; King & Pienaar, 2011). The spatial scope of the RDM approach includes the 19 WMAs selected for South Africa using catchment boundaries (King & Pienaar, 2011). The WMAs for South Africa have since changed to a total of nine WMAs (DWS, 2016a). These WMAs are divided into subcatchments or integrated units of analysis (IUAs) that have a characteristic resource use or development scenarios so that one WRCS class is appropriate for the whole IUA. The WRCS includes stakeholder contributions to choose a high protection, use (but sustainable) of balanced vision for parts of a water resource into: Class I‐ Minimally used; Class II—Moderately used; or Class III—Heavily used (King & Pienaar, 2011). RQOs for water quality, quantity, habitat, and biota are determined to meet the class in an IUA and selected for multiple resources (rivers, estuaries, wetlands, lakes, and groundwater resources) within IUAs. The “National Aquatic Ecosystem Health Monitoring Programme (NAEHMP): River Health Programme (RHP) Implementation Manual.” *Department of Water Affairs and Forestry, Pretoria, South Africa* (2008).

**Fig 2:**
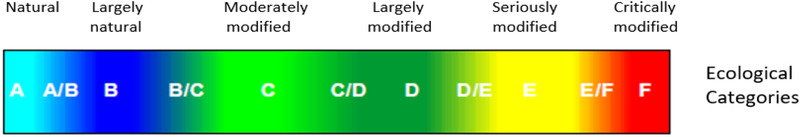
Ecological classification of different components of the RQOs is based on the ecological categories along a continuum. (Adapted from Kleynhans & Louw, 2007.) The corresponding colors are used for easy reference when depicting ECs of river reaches on maps.

South Africa employs another line of evidence (LoE)/suite of tools that complements the RDM procedure, which is used to monitor the implementation of the RDM, namely, the ecological classification (or EcoClassification) process within the National Aquatic Ecosystem Health Monitoring Programme (NAEHMP) (Dallas et al., 2008). The EcoClassification LoE is used to determine the present ecological health relative to the natural reference conditions (Dallas et al., 2008). This guides the understanding of possible sources or causes of deviation from the natural or reference state. The EcoClassification process is used to determine the EcoStatus, which considers a suite of features and characteristics of a water resource, each of which is assigned to an ecological category, based on the deviation from the reference state. Ecological categories are defined as follows: A—Unmodified, natural; B—Largely natural; C—Moderately modified; D—Largely modified; E—Seriously modified; F—Critically modified (Fig. 2) (Kleynhans & Louw, 2007). RQOs once determined are categorized according to the ecological categories A–F. For example, the EC for a specific river reach could be B for fish; C for Invertebrates and C for water quality. Each of these will be accompanied by a narrative, detailing specifications for subcomponents (e.g., water quality specifications may be linked to salt levels or turbidity as legislated requirements for specific uses, which may not be exceeded).

The RQOs are determined using a procedure developed for this specific purpose, involving seven steps, including adaptive management (DWA, 2011). The determination of RQOs is strongly based on risk to the ecological integrity of natural systems and risk to society, with the emphasis on the characteristic features of each IUA. This lends itself to application of BNs, to model the causal pathways between stressors and receptors (exposure pathway) and between receptors and endpoints (effect pathway) (Wade et al., 2021). Critical to this would be the Conditional Probability Table (CPT). It is the weighting or ranking of the input or parent nodes that will determine the effect on the endpoint. Through stakeholder input during the “Visioning” process, the relative values attributed to the water resource by the representatives of different sectors of society are considered. The determination of RQOs is therefore based on consensus and this lays the foundation of agreed priorities to guide water allocation and licenses to authorize water use (DWA, 2011).

**Fig 3:**
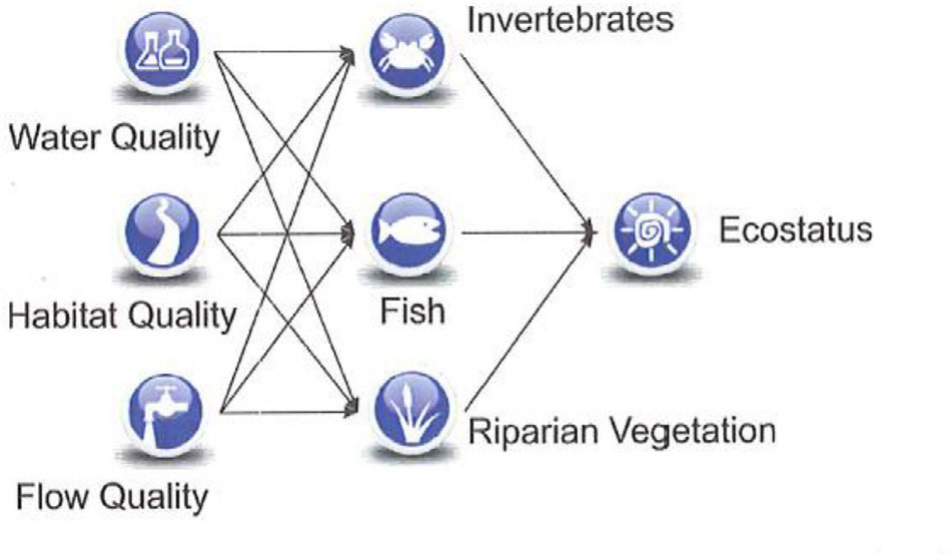
Basic conceptual model representing the variables in the River Health Programme, South Africa (DWS, 2016b).

Adaptive management is a systematic or structured approach, incorporating “learning by doing” and refining practices in response to results of monitoring and evaluation, using structured feedback (Allen, Fontaine, Pope & Garmestani, 2011; Westgate, Likens, & Lindenmayer, 2013). Adaptive management is an iterative process and ultimately serves to facilitate decision making. In applying BNs to ecological models, updating a model through new data or information allows for a system to be regularly revisited. In the South African context, this could be linked to routine monitoring of water resources, locally, regionally, or nationally (Agboola et al., 2020). An example of this is the RHP (DWAF, 2002), which involves sampling of selected biophysical features of water resources to determine the ecological state of rivers in South Africa (Fig. 3). This program involves monitoring carried out twice annually and the results are suitable for modeling using BNs. Through modeling ecosystem variables and their interactions, data deficiencies and gaps in knowledge are being identified. This provides the opportunity to inform monitoring and evaluation programs. Such an approach has the potential to play a significant role in the adaptive management cycle, facilitating continuous updating to optimize management systems.

The graphic nature of BNs facilitates communication of the causal links in socioecological systems, enabling stakeholders and decisionmakers to understand content specific to the scientific discipline (Nojavan, Qian, Paerl, Reckhow, & Albright, 2014). This is especially important in developing countries, when faced with varying literacy levels and in a South Africa, with 11 official languages

### 6.1 BNs to Determine the Limits of the System Endpoints

**Fig 4:**
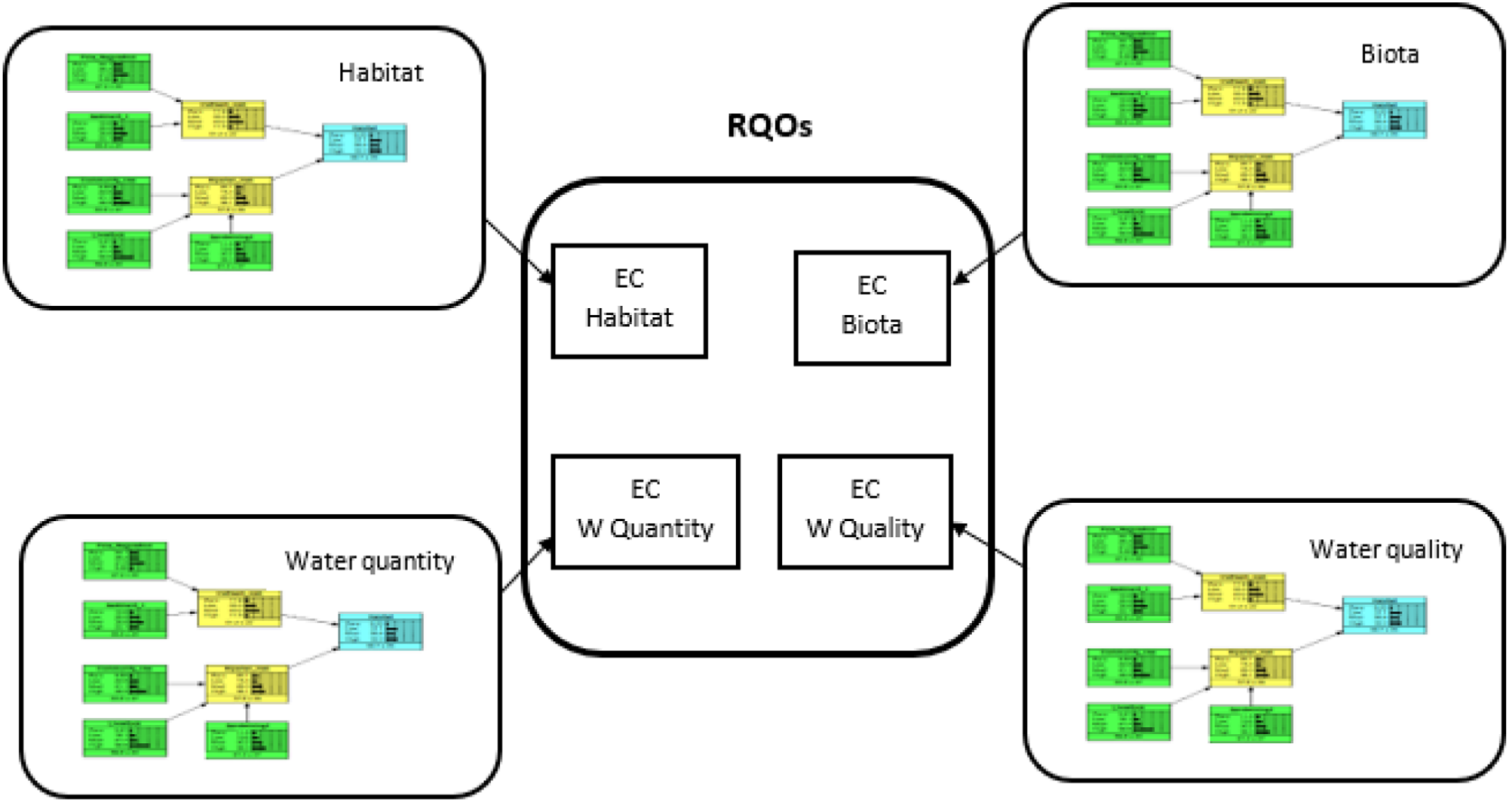
Framework for determination of RQOs through BN application. Each RQO component is represented by a BN submodel, which considers criteria as per the South African EcoClassification indices, used in the current RQO determination process. Specified criteria as outlined in the indices provide the parent node data. The endpoint of each submodel may be linked to ECs.

Endpoints are attributes of a socioecological system that are prioritized for management based on how specific aspects of the system are valued by society (Leuven & Poudevigne, 2002). The potential value of BNs in determining RQOs lies in the power of the CPTs to be configured based on the input of stakeholders and scientists, considering both data and knowledge, as well as societal values placed on the relative importance of use versus protection of water resources. Catchments are unique due to the varying character of each, but there can be rules established for the four components of RQOs: water quality, water quantity, habitat, and biota. Each of these can be represented by a submodel as shown in Fig. 4. The current EcoClassification approach uses different indices to classify the various components of the RQOs. These indices employ metrics, ratings, and rankings of specific criteria that lend themselves to rankings and weightings to be potentially written into a CTP computation. This would be able to present the probability distribution of the endpoint, linked to the ECs. For example, when considering riparian vegetation, the index used for EcoClassification is the Riparian Vegetation Response Assessment Index (VEGRAI) (Kleynhans, MacKenzie, & Louw, 2007). This index is used in conjunction with a spreadsheet model. Criteria such as vegetation cover, vegetation type, response to flows, vegetation removal (for different uses), grazing and human disturbance, and invasive species may constitute the parent nodes in a BN to represent the riparian component of the habitat submodel.

### 6.2 BNs to Determine the Limits of the System Inputs

Alternatively, BNs also have the potential to be used to determine the limits in terms of modification of socioecological systems. When RQOs have been determined for a catchment, the endpoint in a BN may represent the risk distribution for that specific RQO entity (e.g., water quality, biota, etc.). BNs have the ability to determine the distribution of input variables for a specific outcome or endpoint by projecting backward inferences (Ayre & Landis, 2012). Once the BN model has been constructed, the level of stressors can be determined through backward inference, after the specific outcome or endpoint is provided (Agboola et al., 2020; McCann et al., 2006). The risk level for the RQO can be set corresponding to the appropriate EC, followed by backward inference. This would inform the acceptable levels of input variables, which has invaluable practical application for managing sources and stressors.

## 7 CONSIDERATIONS IN BN APPLICATIONS FOR WATER RESOURCES MANAGEMENT IN THE SOUTH AFRICAN CONTEXT

As a developing country with very different needs and priorities compared to Europe and USA, where there has been extensive applications of BNS in catchment‐scale water resources management (Ayre & Landis, 2012; Nojavan et al., 2014; Sperotto et al., 2019; Weil et al., 2018), the question needs to be posed as to whether we are applying models that are adequately “Africanized” to address the issues in South Africa (Varis & Lahtela, 2002). The challenges faced by South Africa related to natural resources management and specifically water resources management are very different from that in the developed world. There are communities relying on direct use of water from rivers, as well as inadequate water and sanitation services in some areas. In addition, expanding urban areas contributes to poor water quality through informal settlements and poor governance (DWAF, 2003; STATSSA, 2019).

The considerations discussed below are characteristic of South Africa, but may be common to other countries globally. Many of these issues are social or socioeconomic but they are very closely linked to the ecology of natural systems as we are very dependent on natural resources and water is a determining factor, or rather a limiting factor, in development. In applying BNs to water resource problems in South Africa, the issues below should ideally be developed into submodels (as described for the RQOs in Fig. 4), which then contribute to the construction of a large model representative of a complex socioecological system. The construction of smaller models may also circumvent the issue raised by Marcot (2017), of huge models that have too many parent nodes that connect to a single child node or limited number of child nodes, resulting in a very complicated CPT. Causal pathways, depicting the links between variables in a system, become explicit and may be easier to understand, when constructing submodels.

### 7.1 Community Engagement in Holistic Water Resources Management

Stakeholders are essential for water management, especially in determining the values placed on water resources to achieve the balance between sustainable use and protection of this vital resource. It is critical that community stakeholders are adequately equipped with the knowledge to play a meaningful role in managing water resources (Rivers‐Moore, 2016). A clear understanding of the intentions behind management objectives should be made explicit from the onset of community engagement in any process. In developing countries where basic service delivery of water and sanitation are major challenges due to economic and logistical issues, as well as poor governance, it is important to get community involved in processes at the outset. This will foster support for water resources management at all levels, with a wide range of stakeholders. Conflict can be avoided by inclusion of stakeholders at every phase of a project or process. It has become common practice to engage community leaders, often from the youth or women representatives, depending on the needs of the community and the demographics, where collaboration plays a significant role in successful execution of water resource management initiatives (Weaver, O'Keeffe, Hamer, & Palmer, 2019). This important link to local communities can be an important source of knowledge and data to inform models incorporating BNs, for water resource management.

### 7.2 Indigenous Ecological Knowledge

As landscapes are developed, there is a loss of indigenous communities and the knowledge that they possess, which may provide a deep understanding of the local ecology (Aswani, Lemahieu, & Sauer, 2018). South Africa has a large diversity of indigenous knowledge, which varies regionally, and this needs to be integrated into water resources management. Indigenous knowledge may hold important background information to guide restoration efforts, when establishing baseline data and information (Hudson et al., 2016; Uprety, Asselin, Bergeron, Doyon, & Boucher, 2012). This would be treated as another form of expert knowledge; however, it would need to be treated with caution, to ensure that it is reliable and verifiable edge. For example, when determining the causal links between variables in a BN, it is important to understand the response of ecosystem variables to environmental cues. Lay knowledge held by local community leaders and traditional knowledge may be critical in providing information to inform ecosystem modeling in relation to climate change mitigation and adaptation (Mantyka‐Pringle et al., 2017; Scott & Barnett, 2009). There are challenges in terms of the power relations where scientific knowledge and traditional knowledge are used in combination. Perceptions and trust are fundamental considerations in such situations (Weingart & Guenther, 2016). If mutual respect and a participatory approach is adopted to build capacity in both researchers/experts (scientific knowledge) and local communities (traditional knowledge), through knowledge exchange, this will go a long way to achieve success in providing expert knowledge to inform BNs. Tribal authorities play a vital role in land issues in South Africa, and this would be critical in land development that may have implications for water resources management (Kapfudzaruwa & Sowman, 2009). Inclusion of this type of knowledge has the potential to play a vital role in the long‐term protection and sustainable use of water resources, as it can pave the way for mutual respect between local communities, government, and scientists, to foster successful collaborative networks.

### 7.3 Ecosystem Services

A well‐functioning ecosystem is fundamental to ecological and social well‐being. Humans are an integral part of natural systems due to our dependence on these systems. In essence, the structure and processes in systems that are intact, support society's requirements, and sustain healthy ecosystems (Munns Jr et al., 2009). Although ecosystem services are most often embodied as endpoints in a BN (where these are benefits derived by humans from nature), there are more specific intangible benefits that should be considered in BNs models. Recent studies on water resources management using BNs are incorporating ecosystem services and disservices (Crossman & Pollino, 2018; Fox, Medina‐Cetina, Angerer, Varela, & Ryang Chung, 2017; Xue, Gui, Lei, Zeng,, 2017). Many catchments in South Africa are under pressure from growing anthropogenic activity. Ecosystem services can be quantified, for example, to represent economic value, and this has the potential to be more meaningful to the business, industry, and agriculture sectors. When these sectors constitute major water users, the application of BNs can be used to illustrate the benefits and negative impacts of their water use, and to highlight trade‐offs under different scenarios. In holistic, complex systems, it is critical that BNs applied to decision making represent the priorities of a wide range of stakeholders, to adequately and accurately reflect the state of the system, related to water resources management.

#### 7.3.1 Supporting Services

Supporting ecosystem services form the foundation on which ecosystems are built, and thereby contribute to all other ecosystem services. This includes water cycling, nutrient cycling, groundwater recharge and contributes to the ecological infrastructure that supports built infrastructure (Russi et al., 2013). Land use changes associated with expansion of agricultural and urban development play a significant role in degrading the provision of supporting ecosystem services, with a resulting ripple effect on the entire suite of important ecosystem services provided by water resources. For example, BNs can be used to illustrate the detrimental impact of agricultural run‐off on waste assimilation of water resources and resultant eutrophication of farm dams.

#### 7.3.2 Provisioning Services

The utilization of water and natural products by small‐scale farmers and local communities is very important. Rural communities throughout South Africa still rely on direct water use from water resources for their basic needs. Hence, their perspective on how water resources are managed should be a priority. Many rural and peri‐urban communities use water directly from rivers to water subsistence crops (DWAF, 2003). Apart from the significant impact this has on the living standards and dignity of communities, it impacts on their health. If water quality is poor or unacceptable, this could result in diseases and illness, as well as loss of productivity. Water is used for cleaning and laundry. Consequently, washing done directly in the water resource impacts on the water quality available for use. An understanding of these issues by the communities using the water resources is vital in the development of linkages between variables in a socioecological system. BNs can be applied to various scenarios to assess management interventions.

#### 7.3.3 Cultural Services

South Africa is a country of diverse cultures, where water plays an important role, especially symbolizing purity and cleansing in religious and cultural rituals. Locality may be related to access to a water resource or due to a specific cultural or religious value attached to the location. Rivers are significant in performing baptisms, initiations, and removal of bad luck (Kapfudzaruwa & Sowman, 2009). Research revealed that traditional governance related to water resources still influences people's views of how water resources are valued and managed. Traditional leaders still play a significant role in the communities that they lead in South Africa. It is therefore beneficial to effective water management that traditional or indigenous governance structures are considered along with relevant local, regional, and national government structures (Kapfudzaruwa & Sowman, 2009).

Many recreational and tourism activities are based around water resources in South Africa. Angling, canoeing, swimming, and nature‐based tourism contribute considerably to the economy in some catchments. Events attracting international participants, such as annual canoeing and swimming events, are significant enough to prioritize protection of water resources. Both international and local tourists are drawn to ecotourism destinations linked to water resources. However, multiple stressors in catchments that impact negatively on water quality may be modeled using BNs to assess risks to various tourism‐related activities. Consequently, the economic benefits can be assessed holistically against the risks to socioecological systems.

#### 7.3.4 Regulating Services

Many communities in South African catchments are located in low‐lying areas, making them vulnerable to flood risk. This is especially true for informal settlements. A risk‐based approach, employing BNs, has the potential to model flood events, especially considering the contribution of climate change to increased flood risk due to increasing rainfall in certain areas of the country. This will help in planning adaptive responses to such events. Working together with communities in addressing long‐term solutions for adaptive response to extreme events associated with climate change, through education and capacity building, is important. This could be enhanced by partnering with other initiatives led by government and nongovernmental organizations (NGOs).

### 7.4 Communicating Effectively

Levels of education still vary in South Africa (STATSSA, 2019), which makes it important that catchment systems are represented in a manner that is easily understood by various stakeholders. BNs have the advantage of being visually explicit in terms of representing system variables. The visual communication potential of BNs through simple conceptual models can make a major and lasting impression on stakeholders, as illustrated in Fig. 3 (Chen & Pollino, 2012). Rivers‐Moore (2016) highlighted the value of using BNs as a participatory tool to engage stakeholders in seeking solutions to complex environmental management problems. However, we must be mindful of the significant impact of language. For example, scientific language and that which is familiar to professionals in a given discipline may be very different from local languages and this could be misinterpreted as hidden agendas to deliberately mislead the community. Fostering trust in engagements between scientists or academics and the local communities is critical to ensuring successful collaborative interactions, especially when dealing with natural resources (Weaver et al., 2019).

Through the process of engaging stakeholders in environmental management projects, knowledge is not only generated but there is also knowledge exchange (Reed, Stringer, Fazey, Evely, & Kruijsen, 2014). Knowledge exchange depends on context and should be a process that continues beyond the life of a project. Throughout the knowledge exchange process, knowledge must be kept relevant and of significance to stakeholders, so that they become a part of the process and their input in model development is meaningful. This will engage stakeholders and not give the impression of providing knowledge and information that is external to their context.

### 7.5 Sustainability Indicators

Like many African nations, South Africa is faced with many challenges, most importantly poverty and inequality. Since the move to a democratic government in 1994, South Africa has made considerable progress, but there is still a great deal to be achieved. Social transformation to meet the basic needs of people promotes economic development, and develops education and training as key priorities. The sustainable development Goals 2030, which addresses development, transformation, and people's dignity, are closely aligned with the South African National Development Plan, in addressing poverty elimination and inequality (Dickens et al., 2019; Morris, 2019) , among other critical issues that are related to water resources management (STATSSA, 2019). With the sustainability agenda a priority in the South African context, BNs provide an opportunity to model water resources in terms of sustainability indicators. The probability of achieving sustainable development goal (SDG) 6, “Ensure availability and sustainable management of water and sanitation for all,” can be modeled using BNs, incorporating an adaptive management approach, to track progress (Dickens et al., 2019). A BN model with variables representing the stressors influencing achievability of SDG 6 using sustainability indicators such as “access to water,“ “access to sanitation,” “households with tapped water,” as endpoints, has the potential to track progress in achieving SDG 6 and other SDGs.

Dickens et al. (2019) proposed a procedure to set targets for natural resources management, aligned with achieving the SDGs. This procedure is based on the seven‐step South African national RQO determination procedure (discussed in Section 6) (DWA, 2011). Multiple spatial scales can be considered in this procedure, including local, regional, and national. Context‐specific socioeconomic character of an area may be considered, making it locally relevant. The adaptive management nature of the procedure makes it conducive to the application of BNs, since data and expert knowledge can be added as progress is made toward the SD targets. The value of this approach may lie in assessing trade‐offs in terms of the ecological and social costs of achieving SD targets. For example, what would be the ecological cost of not achieving the target of providing adequate sanitation to certain areas. This is pertinent to service provision in both formal and informal residential areas, where raw sewage impacts on aquatic ecosystems. The social cost of poor service provision in this case is related to human dignity.

## 8 CHALLENGES IN BAYESIAN NETWORK MODELING OF SOUTH AFRICAN WATER RESOURCES

### 8.1 Simplification of Complex Systems

The flexibility with BNs to be able to “model anything” comes with a cost. There is the potential for overcomplexity in the network structure related to the management problem (Barton et al., 2008). Despite the ability of ERA to consider multiple stressors and represent complex socioecological systems, when applying BNs to complex systems, the greater the number of input variables to a child node, the more complicated the CPTs. As the number of variables in a model increases, more data may be required to support the parameterization process. Too many parent nodes result in large, highly complex CPTs, which become exceedingly difficult to work with (Marcot, 2017). Hence, this may necessitate the simplification of the system to accommodate more practical CPTs. This implies the prioritization of certain variables over others, where previously a model may have been able to accommodate all input variables (Landis, 2021). However, according to Marcot (2017), combining some of the parent nodes and including intermediate nodes may contribute to a better representation of probabilistic dependencies and simplify the CPTs.

Although parts of BNs can be hidden to facilitate communication, it is sometimes necessary to simplify a complex system, when applying BNs to model such a system, to allow for a more manageable model, particularly where multidisciplinary input is required. If so, it is important to specify how a complex system is simplified for representation by a model. On the other hand, it is important that the BN can capture relevant dynamics. Some types of BNs have difficulty in dealing with feedback loops, which often are needed when representing ecological systems (Aguilera et al., 2011; Hart & Pollino, 2008). If details are omitted from a model, it could lead to a very different outcome to what is anticipated. This would be a critical consideration when using BNs to represent complex catchment for regulatory purposes, such as RQO determination in South Africa.

The majority of software for BNs are designed for categorical nodes to represent the system. Using categorical or discretized continuous variables improves efficiency when running the model. State variables in natural systems are most often continuous, and discretization of continuous variables, may result in information loss (Uusitalo, 2007). The method employed for discretization may impact on the predictive quality of the model (Ropero, Renooji, & van der Gaag, 2018), and careful consideration must be given to when and how to discretize. For data‐driven BNs without *a priori* distributions, a large number of intervals for a variable can result in zero frequencies and a failure to be adequately representative of the natural system (Uusitalo, 2007). Discretization can alternatively be adaptive, with intervals adjusting to the distribution in data (Koller & Friedman, 2009). Using few categories may, on the other hand, reduce information and limit the possibilities to detect or predict extreme events. The type of BN should primarily be chosen to represent the system at hand and be able to integrate the type of information available, and then appropriate software or original code should be used.

### 8.2 Accounting for Uncertainty

Uncertainty refers to any limitations in knowledge. To ensure trust and transparency when models are informing decision making, it is important to communicate the assessor(s) uncertainty about the conclusions from the assessment. Uncertainty can be described quantitatively or qualitatively and can include caveats or weaknesses in the information supporting the assessment or disagreement of the model and assumptions used for the assessment. As with any model, there are limitations in knowledge that may not be able to integrate into the model and therefore must be communicated qualitatively (Goerlandt & Montewka, 2015; Sahlin et al., 2021). In modeling socioecological systems and the inherent complexities of such systems, the need to account for and quantify uncertainty is a critical issue, in order to ensure sound management of natural resources and practically implementable solutions to environmental problems (Ascough, Maier, Ravalico, & Strudley, 2008).

BNs can quantify the impact of some sources to uncertainty, but not all (Sahlin et al., 2021). As in all scientific models, it is important to reduce uncertainty about what the model represents. This can be done by using unambiguous definitions of the nodes, and make sure that the experts understand them, and be clear what the BN joint probability distribution represents. The assessor must be clear if the BN is a model of the random behavior of the system, also known as aleatory uncertainty or variability, or if it is a model of their uncertainty about the system, also known as epistemic uncertainty (Abdo & Flaus, 2016; Sahlin et al., 2021). In most environmental applications of BN, and in particular where the nodes are continuous variables randomly taking different values over time, the network is a model of aleatory uncertainty, and the probability distribution is expressing the relative frequencies of events. The probability distribution of the output node is showing how often the output node takes different values over, e.g., time. In other applications, no distinction is made between aleatory and epistemic uncertainty, with the consequence that we cannot say how much of the probability of the adverse event is due to random behavior of the system or due to our lack of knowledge about the system.

The varying practice to distinguish variability from (epistemic) uncertainty in BN is a challenge for applications that can be solved by acknowledging this issue and to find solutions to treat uncertainty (Sahlin et al., 2021). Uncertainty about the model structure can be treated by using multiple BNs. Uncertainty about the probabilities in the network (CPTs) can be treated by studying the influence of alternative choices of CPTs. Sensitivity analysis can be used to determine which are the most influential nodes to indicate where it may be worthwhile to reduce uncertainty (Beaudequin, Harden, Roiko, & Mengersen, 2017).

It is important that the assessors are able to demonstrate the validity of the assessment relying on BNs. As a complement to validating with respect to independent data, which is likely to be lacking for the full system that is being modeled, validation is about showing transparency about the process to build the BN and get information from experts and stakeholders. BNs used for scientific advice should document both data and expert elicitation in a manner that allows a well‐grounded defense if required and supports validation of the model structure and conditional probabilities. Where expert knowledge is the main source of information, testable hypotheses must be developed. In such cases, the model must be tested and updated by following repeatable, valid procedures (Marcot et al., 2006; Sahlin et al., 2021).

## 9 CONCLUSION: BAYESIAN NETWORKS FOR FUTURE SUSTAINABLE WATER RESOURCES MANAGEMENT IN SOUTH AFRICA

In a semarid country such as South Africa, the published literature indicates that BNs are largely unexplored in addressing risks to water resources. In 2012, O'Brien and Wepener presented the RRM, which is internationally applied, as a structured ERA approach to be used in South Africa to model water resources management, with the aim of achieving a balance between the use and protection of water resources. In their publication, they explained the merits of this framework, including the application to unique case studies and conditions in South Africa. Subsequent to this publication, BNs were added to the RRM framework and applied to various studies, further exhibiting the merits of BN applications to complex systems (O'Brien, Dickens, et al., 2018; O'Brien et al., 2020; Wade et al., 2021). One of the benefits this provides is the capability of predicting future scenarios, making it an excellent tool for exploring uncertain futures, such as climate change scenarios.

Based on the vast application of BNs in addressing various environmental problems, using a risk‐based approach to present alternative management scenarios, it is clear that South Africa has the opportunity to explore the merits of this modeling tool. Considering spatial and temporal context and taking cognizance of the unique issues facing water resources management, BNs can provide a valuable means of communicating socioecological interactions to a wide audience. A model presented to stakeholders is most meaningful when stakeholders are given the opportunity to provide input to the model to develop it into an accurate representation of reality, considering all relevant concerns. Such a model can continue to develop and evolve as changes occur in a region or catchment, through a process of adaptive management, as the model is updated. This has the potential to create a blueprint for integration of BN models with existing monitoring programs and processes to foster holistic, sustainable water resources management in South Africa.

In dealing with dynamic socioecological systems that are of significance to society, we may not have the resources to look at all attributes of these ecosystems. Thus, probabilistic tools that are robust yet informative present opportunities to model such systems to enhance or guide decision making. Their ability to explicitly present uncertainty provides regulators and stakeholders with available evidence to guide decision making in an adaptive context to implement policies, and establish resource use and protection requirements. This has the potential to facilitate enforcement of regulations in complex socioecological systems where incomplete empirical data are often a reality. Presently, in South Africa, there is a disconnect between the knowledge that we are overutilizing resources, and our inability to implement globally recognized legislation and policy. Application of BNs can bridge this gap and allow us to manage resources with limited knowledge.

BNs have moved from traditionally being viewed as probabilistic models of categorical nodes informed by experts, to include models for data‐driven networks, to models linking system variables informed by expert judgment, data, or both. Limiting BNs to probabilistic networks with categorical nodes have limitations when modeling natural systems, which must be overcome to use BNs for scientific consultation. Viewing BNs as probabilistic graphical models including Bayesian hierarchical statistical models (Varis & Kuikka, 1997) and probabilistic process‐based models (Landis et al., 2020) into the family of BNs is beneficial, and even necessary, for the future use of BNs in ERA and water management. The ability to update and improve the decision‐making process allows BNs to be truly adaptable, which has been established as good international practice. The use of BNs, especially within the ERA context, has globally been demonstrated to be a robust approach to model water resources management. These applications to address challenges in socioecological systems in South Africa have the potential to make a noticeable positive contribution to achieving a sustainable balance between the use and protection of our resources in a holistic manner.

## Supporting information

Supplementary information
